# Comparisons of health-related factors in children with attention-deficit/hyperactivity disorder with and without sleep problems following a weighted blanket sleep intervention

**DOI:** 10.1186/s12887-025-06051-3

**Published:** 2025-09-09

**Authors:** Julia S. Malmborg, Josefine Tuvesson, Ingrid Larsson, Petra Svedberg, Jens Nygren, Håkan Jarbin, Annelie Lindholm

**Affiliations:** 1 School of Health and Welfare, Halmstad University, Halmstad, Sweden; 2 Faculty of Medicine, Department of Clinical Sciences, Lund University, Lund, Sweden; 3 Child and Adolescent Psychiatry, Region Halland, Halmstad, Sweden

**Keywords:** Attention-deficit/hyperactivity disorder, Children, Health, Sleep

## Abstract

**Background:**

Adequate sleep is crucial for children’s health, especially for children with ADHD and concurrent sleep problems. There is a need for more studies focusing on sleep problems in children with ADHD as these problems may exacerbate ADHD symptoms and vice versa, impacting negatively on everyday life. The aim of this study was to investigate the differences in health-related factors between children with ADHD without clinically relevant sleep problems and those with clinically relevant sleep problems after a sleep intervention.

**Methods:**

This cross-sectional study involved 83 children diagnosed with ADHD and sleep problems, 46 boys and 37 girls; aged 6–14 years, divided into two groups after a sleep intervention according to the parent-reported Children’s Sleep Habits Questionnaire (CSHQ) and a cut-off for clinically relevant sleep problems. Data from a 16-week follow-up of the sleep intervention were analysed in terms of the following health-related measures: The Insomnia Severity Index (ISI), The short form of State-Trait Anxiety Inventory (Short-STAI), The Child Outcome Rating Scale (CORS), The EQ-5D-Y-3L (child-reported), and The Swanson, Nolan, and Pelham Rating Scale (SNAP-IV) (parent-reported). The Mann-Whitney u-test, Independent samples t-test, and Chi-square/Fisher’s exact test were used for the analysis.

**Results:**

Forty-two of the 83 children (50.6%) were grouped as having clinically relevant sleep problems and 41 (49.4%) as being without the same. The results indicated that the group without clinically relevant sleep problems reported less insomnia (ISI, total score *p* = 0.011), less tension in the anxiety scale (Short-STAI, tense *p* = 0.047), and their parents reported less ADHD symptoms (SNAP-IV, total score for attention deficit, *p* < 0.001). No group differences were observed for life functioning (CORS) or health-related quality of life (EQ-5D-Y-3 L).

**Conclusions:**

This study showed that children with ADHD without parent-reported clinically relevant sleep problems had fewer health-related issues after a sleep intervention, including self-reported insomnia, tension, and parent-reported ADHD symptoms, compared to children with clinically relevant sleep problems. Longitudinal studies are necessary to fully comprehend the long-term impact of sleep problems and various health-related factors in this cohort.

## Background

Attention-Deficit/Hyperactivity Disorder (ADHD) is a neurodevelopmental disorder, characterized by attention deficit and hyperactivity with or without impulsivity and difficulties in managing and controlling emotions and mood [1]. In addition to cognitive and behavioral impairments, children with ADHD have been shown to be afflicted with low sleep quality and increased daytime sleepiness [2]. Insufficient sleep is a major concern for children in general, but especially for children with ADHD [1]. The prevalence of sleep problems has been found to vary between 25% and 85% in children with ADHD [3–5] in comparison with the prevalence of sleep problems of 20–30% in children without neurodevelopmental disorders [6]. Since adequate sleep is crucial for children’s behavior and daily function, it is an important part of children’s overall health [7]. Common sleep problems in ADHD may stem from a hypo-arousal state, from delayed sleep onset latency and thus bedtime resistance, from sleep anxiety, parasomnia [8], sleep disordered breathing [9] and from restless legs syndrome, which is a common comorbidity to ADHD in children and often related to iron deficiency [9–11]. Inadequate sleep among children diagnosed with ADHD is associated with academic challenges [4, 12], poorer mental health and lower quality of life [13].

Numerous studies on sleep problems in children with ADHD have focused on variables such as comorbidities and pharmacological treatments [14–18]. However, some of these have overlooked other important health-related factors such as insomnia, anxiety, and ADHD symptoms as well as overall well-being and how the children function in their everyday life. Understanding the complexity of sleep problems in children with ADHD is crucial, and although studies specifically addressing sleep problems in this group of children have been performed [19, 20] more research is needed.

The aim of this study was to investigate the differences in health-related factors between children with ADHD without clinically relevant sleep problems and those with clinically relevant sleep problems after a sleep intervention.

## Methods

### Study design, recruitment, and participants

The current study had a cross-sectional design and included data collected from a randomized controlled trial with a sleep intervention with weighted blankets [21] targeted towards children aged 6–14 years with ADHD and sleep problems. The participants were recruited between January 2020 and January 2022 when attending an ADHD unit at a Child and Adolescent Mental Health Service (CAMHS) in the south of Sweden. Children were enrolled to the specific ADHD unit because they did not have significant comorbidities or complex social challenges [22]. Those with significant comorbidities or complex social challenges were treated at other units at CAMHS. Children with recently diagnosed ADHD and concurrent sleep problems were considered eligible for inclusion. Children were triaged to the unit when the structured Brief Child and Family Phone Interview indicated a probable diagnosis of ADHD. The assessment at the unit was based on a written report by a teacher at the child’s school regarding strengths and difficulties with learning and social functioning at school, a teacher ADHD rating scale, a thorough diagnostic interview with the child and the parent(s), and an observation of the child. The diagnostic interview was carried out by a resident, and the diagnosis was made based on the DSM-5 criteria and confirmed by a senior child and adolescent psychiatrist at the end of each assessment. Medication was also prescribed in about half of the cases. Sleep problems were screened by a healthcare professional from CAMHS utilizing three questions (scored as 0–1, 2–4, or 5–7 days per week) to the parent from the Swedish version of the Children’s Sleep Habits Questionnaire (CSHQ-SWE) [23, 24], namely: Question 2 (“Child falls asleep within 20 minutes after going to bed”, scoring criteria 0–1 or 2–4 days per week), question 9 (“Child sleeps too little”, scoring criteria 2–4 or 5–7 days per week), and question 25 (“Child awakes more than once during the night”, scoring criteria 2–4 or 5–7 days per week). Each question contained a follow-up question about whether the parents experienced the question’s area as a problem (yes/no). If at least one of the questions met the scoring criteria and that the question’s area was perceived as a problem, the child was considered eligible for inclusion. More information regarding the recruitment process and the representativeness of the sample is described elsewhere [21].

Ethical approval was granted by the Swedish Ethical Review Authority (no. 2019–02158) adhering to guidelines set by the Declaration of Helsinki. Participation was voluntary and participants were informed of their right to withdraw without explanation. Oral and written information about the study was presented and, after receiving information, parents signed informed consents for themselves and on behalf of their children before entering the study.

**Fig. 1:**
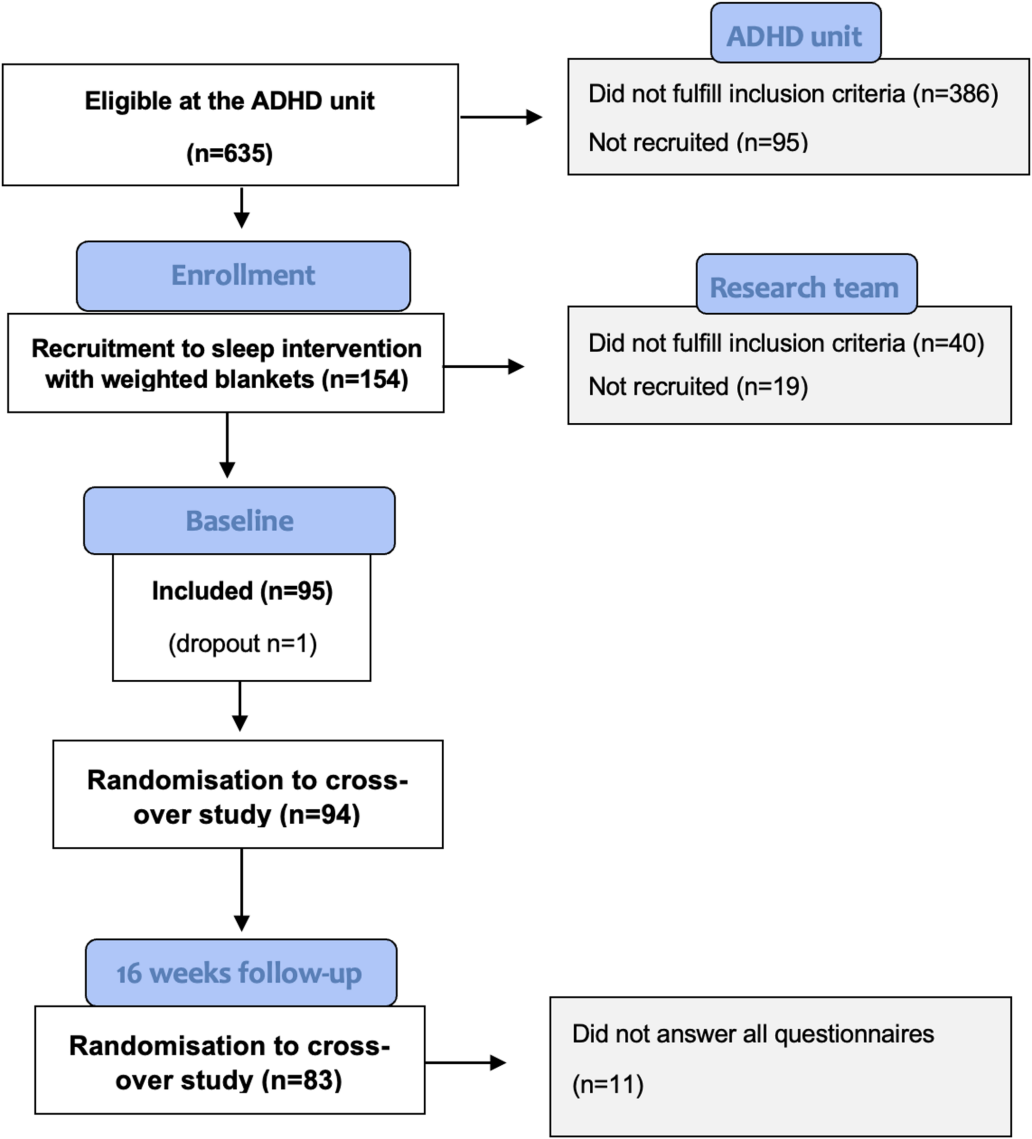
CONSORT flow diagram of the recruitment and patients enrolled in this study

A total of 154 children were considered eligible after diagnostics and screening had been performed. The parents and children were invited by telephone to participate in the research project, of whom 94 child-parent dyads (53 boys and 41 girls) accepted and consented to participate. In the current study, 83 (46 boys and 37 girls, aged 6–14) of the 88 children with parents who completed the 16-week intervention period were included (Fig. 1).

### Intervention and data collection

The sleep intervention with weighted blankets included a cross-over phase (4 + 4 weeks) and an open-label phase (8 weeks). The children were randomized into sleeping with a weighted blanket or a lighter control blanket for the first four weeks of the cross-over phase, and then a cross-over was performed of the weighted blanket group and control blanket group for the following four weeks. The children then slept during the open-label phase with either the weighted blanket or the lighter control blanket depending on which they preferred. The data collection was made through repeated measurements across four measurement weeks: baseline and weeks 4, 8, and 16. Sociodemographic data (age and gender of children, ADHD subtype, medication, use of stimulants, age and gender of parents, educational level, employment status and civil status) were gathered at baseline. The data utilized in this study consisted of the information collected at the 16-week follow-up.

### Health-related measures

#### Children’s Sleep Habits Questionnaire – Swedish version (CSHQ-SWE)

**Fig. 2:**
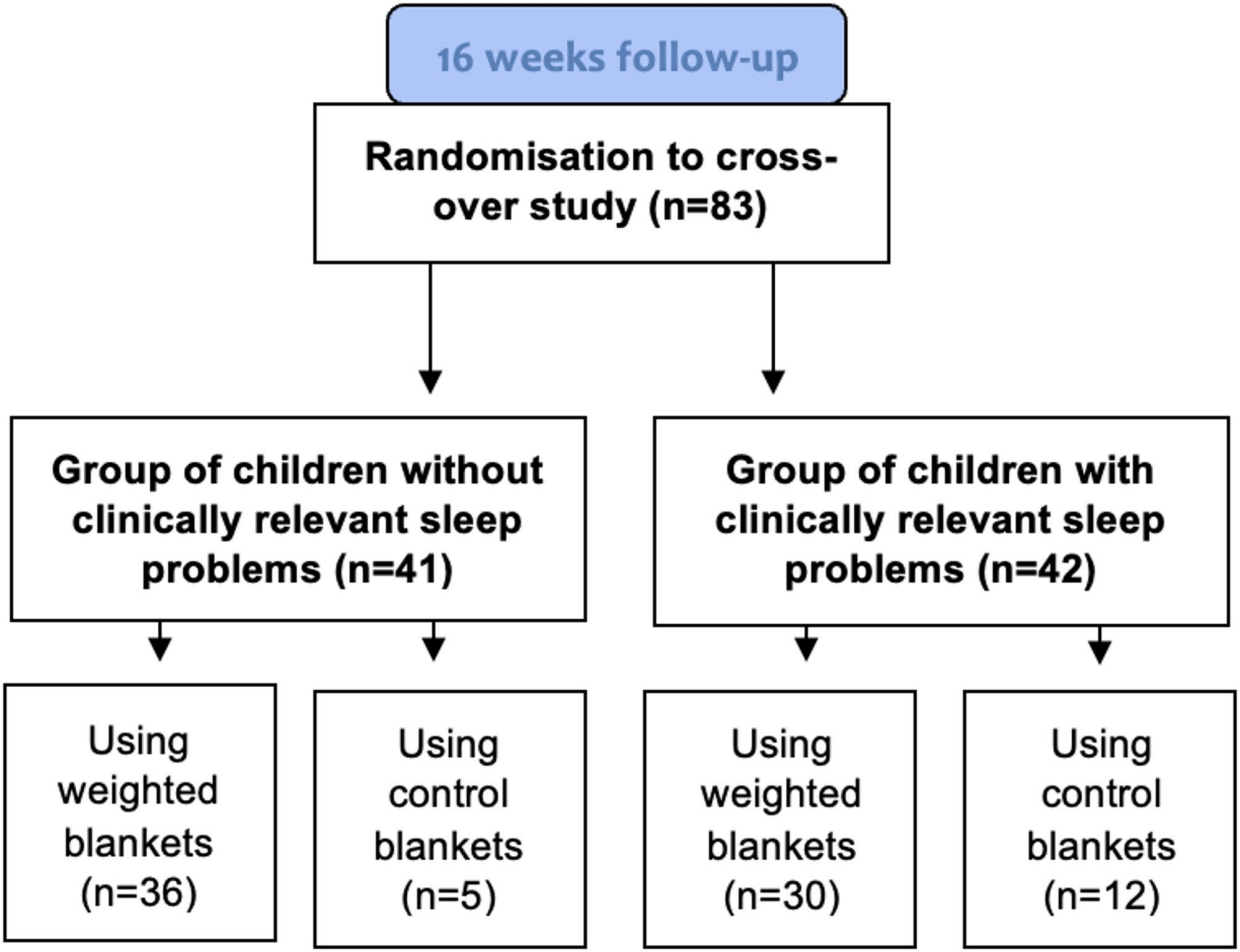
CONSORT flow diagram of the participants in the analyses of this study

Children’s Sleep Habits Questionnaire – Swedish version (CSHQ-SWE) [24] is a questionnaire completed by parents to describe their child’s sleep problems during the preceding week. It is a Swedish version of the English original [23] and has been validated in the target group [24]. The questionnaire consists of 33 items and the total score ranges from 33 to 99. The parents estimate the occurrence of sleep problems on questions 1–31 as “usually” (occurs 5 times or more during a week, 3 points), “sometimes” (occurs 2–4 times per week, 2 points) or “rarely” (occurs never or once a week, 1 point). For questions 32 and 33, sleepiness during various activities is estimated as “not sleepy” (1 point), “very sleepy” (2 points) and “falls asleep” (3 points) [23]. A higher total score indicates greater severity of sleep problems, and the cut-off for clinically relevant sleep problems for children with ADHD is ≥ 48 points [25]. The participants in this study were divided into two groups based on the classification criteria for children with ADHD [25] at the 16-week follow-up: those without clinically relevant sleep problems (CSHQ-SWE total score < 48 points) and those with clinically relevant sleep problems (CSHQ-SWE total score ≥ 48 points). Grouping was performed irrespective of the choice of a weighted blanket or a control blanket during the open-label phase, and irrespective of adherence to chosen blanket (Fig. 2).

#### Insomnia Severity Index (ISI)

Insomnia Severity Index (ISI) is a seven item self-reported measure for children and adolescents used to evaluate insomnia. It covers difficulties falling asleep, maintaining sleep, early morning awakening, sleep satisfaction, interference with daily functioning, noticeability of sleep problems, and distress regarding sleeping problems. Each item is rated on a five-point Likert scale, with scores ranging from 0 to 4 and the total score is in the range 0–28. Higher scores on the ISI reflect increased severity [26]. The measure in this study was modified with regards to language to enhance applicability and comprehension among younger users, ensuring that it resonated with their developmental stage and cognitive abilities.

#### The short form of State-Trait Anxiety Inventory (short STAI)

The short form of State-Trait Anxiety Inventory (short STAI) is a six-item self-reported measure for children and adolescents for evaluating anxiety. The items Calm, Tense, Upset, Relaxed, Content, and Worried are each rated on a four-point Likert scale. The total score ranges from 6 to 24 points, with higher scores indicating higher anxiety levels [27, 28]. The short STAI is used when the full form is considered to be too comprehensive.

#### Child Outcome Rating Scale (CORS)

The self-reported Child Outcome Rating Scale (CORS) for children in the ages 6–12 years assesses life functioning across four domains including own well-being (How am I doing?), family (How are things in my family?), school (How am I doing at school?), and overall well-being (How is everything going?) on a visual analogue scale from 0 (worst, frowny face) to ten (best, happy face). In addition, it contains an overall total score, which ranges from 0 to 40 points (worst to best), with higher scores indicating better life functioning [29].

#### EQ-5D-Y-3 L

EQ-5D-Y-3 L is a five-item self-reported measure for children and adolescents used to evaluate health-related quality of life. The measure includes the following domains: mobility, looking after myself, doing usual activities, having pain or discomfort, and feeling worried, sad, or unhappy. Each domain is answered on a scale from 1 to 3, where 1 corresponds to no problems, 2 corresponds to some problems, and 3 indicates significant problems. Additionally, a visual analogue scale (VAS) was used. The VAS ranges from 0 to 100, reflecting overall health, with a higher score indicating better perceived health [30, 31].

#### The Swanson, Nolan, and Pelham Rating Scale – Parent-reported version (SNAP-IV parent)

SNAP-IV parent, which assesses symptoms of ADHD, comprises 18 items on a four-point Likert scale (ranging from 0 = ‘not at all’ to 3 = ‘very much’) to assess the severity of symptoms. Nine items cover attention deficit, six items hyperactivity, and three items impulsivity, yielding 0–27 points for attention deficit and for hyperactivity/impulsivity respectively. A combined measure yields 0–54 points. Higher scores indicate a heightened manifestation of ADHD symptoms [32].

### Data analysis

The Shapiro Wilks test was used to analyse normal distribution of variables. Sociodemographic data and outcome measures (ISI, Short-STAI, CORS, EQ-5D-Y-3 L, EQ-5D-Y-3 L VAS, and SNAP-IV parent) were presented as means and standard deviations (SD), medians and interquartile ranges (IQR), or numbers and percentages. Due to limited distribution in answers, the response options of “some problems” and “significant problems” were merged and categorized as “problems” in EQ-5D-Y-3 L. Analyses between the groups with and without clinically relevant sleep problems were conducted by independent samples t-tests or by Mann-Whitney U tests, depending on normal distribution of data, and by Chi-square tests (Fisher’s exact test or Fisher-Freeman-Halton exact test where appropriate) for categorical variables. Effect sizes for independent samples t-tests were analysed with Cohen’s d (d = 0.20–0.49 representing small, d = 0.50–0.79 representing medium, and d ≥ 0.80 representing large [33] and for Mann-Whitney U tests with r (*r* < 0.3 representing small, *r* = 0.3–0.5 representing medium, and *r* > 0.5 representing large). IBM SPSS Statistics for Windows version 28 (IBM Corp, Armonk, New York, USA) were used and p-value was set to *p* < 0.05.

## Results

The study consisted of 83 participants (45 boys and 38 girls; 6–14 years old). At the 16-week follow-up, 41 of these were grouped as being without parent-reported clinically relevant sleep problems (23 boys and 18 girls) and 42 were grouped as having parent-reported clinically relevant sleep problems (22 boys and 20 girls). There were no differences in sociodemographic factors between the two groups at baseline (Tables 1 and 2).

**Table 1:** Comparisons of sociodemographic baseline factors for children between the groups at the 16-week follow-up

	No clinically relevant sleep problems, *n* = 41	Clinically relevant sleep problems, *n* = 42	U/t	z	*p*-value
***Children***					
**Age, mean ± SD**	9.54 ± 2.12	9.55 ± 2.43	−0.022		0.982
**Age, median (IQR)**	9.00 (8.00–11.00)	9.50 (7.00–12.00)	856.50	−0.041	0.967
Age, 6–9 years (%)	22 (53.7)	21 (50.0)			0.827
Age, 10–14 years (%)	19 (46.3)	21 (50.0)			
**Gender**					
Male (%)	23 (56.1)	22 (52.4)			0.827
Female (%)	18 (43.9)	20 (47.6)			
**ADHD diagnosis**					
Attention deficit (%)	13 (31.7)	10 (23.8)			0.469^a^
Hyperactivity and impulsivity (%)/Combined (%)	1 (2.4)/27 (65.9)	1 (2.4)/31 (73.8)			
**Medication**					
No medication (%)	21 (51.2)	24 (57.1)			0.662
Medication (%)	20 (48.8)	18 (42.9)			
**Stimulants**					
No stimulants (%)	23 (56.1)	20 (47.6)			0.512
Stimulants (%)	18 (43.9)	22 (52.4)			

Results are expressed as means and SD, median and IQR, or number and percentage. Independent samples t-test and Mann-Whitney U test were used to analyse differences in children’s mean ages. Comparisons of children’s age groups, gender, ADHD diagnosis, medication, and use of central stimulants were conducted using Chi-square test or Fisher’s exact test

^a^Analysed as attention deficit vs. hyperactivity, impulsivity and combined

ADHD, attention-deficit/hyperactivity disorder; IQR, interquartile range; SD, standard deviation

**Table 2:** Comparisons of sociodemographic baseline factors for parents between the groups at the 16-week follow-up

	No clinically relevant sleep problems, *n* = 41	Clinically relevant sleep problems, *n* = 42	U/t	z	*p*-value
***Parents***					
**Age, mean ± SD**	39.41 ± 5.55	39.05 ± 5.50	0.303		0.763
**Age, median (IQR)**	40.00 (35.50–42.00)	38.00 (35.75–42.25)	799.00	−0.566	0.571
Age 28–39 (%)	20 (48.8)	26 (61.9)			0.273
Age 40–52 (%)	21 (51.2)	16 (38.1)			
**Gender**					
Male (%)	5 (12.2)	3 (7.1)			0.483^a^
Female (%)	36 (87.8)	39 (92.9)			
**Educational level**					
Elementary school (%)	2 (4.9)	4 (9.5)			0.480^b^
High school (%)	17 (41.5)	13 (31.0)			
University (%)	22 (53.7)	25 (59.5)			
**Employment** ^c^					
Employed (%)	38 (92.7)	34 (81.0)			0.194
Other (%)	3 (7.3)	8 (19.0)			
**Civil status**					
Lives alone (%)	5 (12.2)	8 (19.0)			0.548
Lives with a partner (%)	36 (87.8)	34 (81.0)			

Results are expressed as means and SD, median and IQR, or number and percentage. Independent samples t-test and Mann-Whitney U test were used to analyse differences in parents’ mean ages. Comparisons of parents’ age groups, gender, educational level, employment status, and civil status were conducted using Chi-square test or Fisher's exact test

^a^ Fisher’s exact test

^b^ Fisher-Freeman-Halton exact test

^c^ Employment was grouped as employed (full time or part time) or other (unemployment, parental leave, studying or sick leave)

IQR, interquartile range; SD, standard deviation

The children without clinically relevant sleep problems scored better in the analysis of ISI in terms of the variables falling asleep, satisfaction, and the total score compared to the group that had clinically relevant sleep problems. No group differences were observed in the remaining subscales (Table 3). The group without clinically relevant sleep problems scored better for the subscale tense in the Short-STAI, but none of the other variables revealed any differences between the groups (Table 3).

**Table 3:** Comparisons of ISI and Short-STAI between the groups at the 16-week follow-up

	Median (IQR Q1–Q3)		U	z	*p*-value	Effect size *r*
	No clinically relevant sleep problems, *n* = 41	Clinically relevant sleep problems, *n* = 42				
**ISI (Child-reported)**						
Falling asleep (0–4)^a^	1.00 (1.00–1.50)	1.50 (1.00–2.00)	628.50	−2.242	**0.025**	−0.246
Maintaining sleep (0–4)^a^	0.00 (0.00–1.00)	0.50 (0.00–1.00)	837.50	−0.237	0.812	−0.026
Early morning awakening (0–4)^a^	0.00 (0.00–1.00)	0.50 (0.00–2.00)	718.00	−1.461	0.144	−0.160
Satisfaction (0–4)^a^	0.00 (0.00–1.00)	1.00 (0.00–1.00)	649.00	−2.171	**0.030**	−0.238
Interference (0–4)^a^	1.00 (0.00–2.00)	1.00 (1.00–2.00)	730.50	−1.233	0.218	−0.135
Noticeability (0–4)^a^	1.00 (0.00–2.00)	1.00 (0.75–2.25)	821.50	−0.371	0.711	−0.041
Distress (0–4)^a^	0.00 (0.00–1.00)	0.50 (0.00–1.00)	711.50	−1.537	0.124	−0.169
Total score (0–28)^a^	5.00 (3.00–8.00)	7.50 (5.75–10.25)	583.50	−2.537	**0.011**	−0.278
**Short-STAI (Child-reported)**						
Calm (1–4)^a^	2.00 (1.00–2.00)	2.00 (1.00–2.00)	760.00	−0.997	0.319	−0.109
Tense (1–4)^a^	1.00 (1.00–2.00)	2.00 (1.00–2.00)	671.00	−1.986	**0.047**	−0.218
Upset (1–4)^a^	1.00 (1.00–2.00)	1.00 (1.00–2.00)	772.00	−1.008	0.313	−0.111
Relaxed (1–4)^a^	2.00 (1.00–2.00)	2.00 (1.00–2.00)	859.00	−0.200	0.984	−0.022
Content (1–4)^a^	2.00 (1.00–2.00)	2.00 (1.00–2.00)	722.00	−1.431	0.152	−0.157
Worried (1–4)^a^	1.00 (1.00–1.50)	1.00 (1.00–2.00)	816.00	−0.532	0.594	−0.058
Total score (6–24)^a^	9.00 (7.00–10.50)	10:00 (8:00–12:00)	718.50	−1.309	0.191	−0.144

Note: p-values of < 0.05 are marked in bold

Results were analysed with Mann-Whitney U test (effect size r) and presented as median and IQR

^a^Total possible ranges for ISI and Short-STAI, scored best–worst

ISI, Insomnia Severity Index; IQR, interquartile range; Q, quartile; Short-STAI, The short form of State-Trait Anxiety Inventory

The findings from CORS indicated that there were no statistically significant differences between the two groups of children in the total score or in any of the sub domains (Table 4).

**Table 4:** Comparisons of CORS between the groups at the 16-week follow-up

	Median (IQR Q1–Q3)		U	z	*p*-value	Effect size *r*
	No clinically relevant sleep problems, *n* = 41	Clinically relevant sleep problems, *n* = 42				
** CORS (Child-reported)**						
Me (0–10)^a^	9.30 (7.60–10.00)	8.10 (6.25–10.00)	664.00	−1.827	0.068	−0.201
Family (0–10)^a^	10.00 (8.85–10.00)	9.30 (7.53–10.00)	718.00	−1.370	0.171	−0.150
School (0–10)^a^	8.50 (7.00–9.85)	7.65 (5.25–9.45)	739.00	−1.116	0.265	−0.122
Everything (0–10)^a^	9.10 (7.40–10.00)	8.90 (6.98–10.00)	729.50	−1.220	0.223	−0.134
Total score (0–40)^a^	36.00 (30.35–38.80)	33.10 (27.78–36.45)	665.50	−1.782	0.075	−0.196

Results were analysed with Mann-Whitney U test (effect size r) and presented as median and IQR

^a^Total possible ranges for CORS, scored worst–best

CORS, Child Outcome Rating Scale; IQR, interquartile range; Q, quartile

The result of the five dimensions in the EQ-5D-Y-3 L showed no statistically significant results between the two groups of children (Table 5), and there were no statistically significant differences in perceived health observed in the EQ-5D-Y-3 L VAS (Table 6).

**Table 5:** Comparison of EQ-5D-Y-3 L between the groups at the 16-week follow-up

	No clinically relevant sleep problems, *n* = 41 (%)	Clinically relevant sleep problems, *n* = 42 (%)	*p*-value
**EQ-5D-Y-3 L (Child-reported)**			
**Mobility**			
No problems	40 (97.6)	40 (95.2)	1.000^a^
Problems^b^	1 (2.4)	2 (4.8)	
**Looking after myself**			
No problems	38 (92.7)	32 (76.2)	0.067
Problems^b^	3 (7.3)	10 (23.8)	
**Doing usual activities**			
No problems	38 (92.7)	34 (81.0)	0.194
Problems^b^	3 (7.3)	8 (19.0)	
**Having pain or discomfort** ^c^			
No problems	35 (87.5)	31 (73.8)	0.165
Problems^b^	5 (12.5)	11 (26.2)	
**Feeling worried, sad, or unhappy**			
No problems	33 (80.5)	28 (66.7)	0.214
Problems^b^	8 (19.5)	14 (33.3)	

Results were analysed with Chi-square test or Fisher’s exact test and presented as number and percentage

^a^Fisher’s exact test

^b^Options “some problems” and “significant problems” were merged and categorized as “problems”

^C^One person missing in domain from the group of no clinically relevant sleep problems

**Table 6:** Comparisons of EQ-5D-Y-3 L VAS between the groups at the 16-week follow-up

	Median (IQR Q1–Q3)		U	z	*p*-value	Effect size *r*
	No clinically relevant sleep problems, *n* = 41	Clinically relevant sleep problems, *n* = 42				
**EQ-5D-Y-3 L (Child-reported)**						
VAS (0–100)^a^	93.00 (80.00–100.00)	89.50 (75.00–96.25)	720.50	−1.291	0.197	−0.142

Results were analysed with Mann-Whitney U test (effect size r) and presented as median and IQR

^a^Total possible range for EQ-5D-Y-3 L VAS, scored worst–best

IQR, interquartile range; Q, quartile; VAS, visual analogue scale

Analyses from SNAP-IV revealed that the group without clinically relevant sleep problems displayed a lower severity of attention deficit in comparison with the other group, as well as a lower severity of the combined score for attention deficit and hyperactivity/impulsivity (Table 7). 

**Table 7:** Comparisons of SNAP-IV parent between the groups at the 16-week follow-up

	No clinically relevant sleep problems, *n* = 41	Clinically relevant sleep problems, *n* = 42	t	*p*-value	Effect size Cohen’s d
**SNAP-IV (parent-reported)**					
Total score attention deficit (0–27)^a^, mean ± SD	13.24 ± 4.72	17.74 ± 4.88)	−4.265	**< 0.001**	−0.936
Total score hyperactivity and impulsivity (0–27)^a^, mean ± SD	12.20 ± 6.19	14.38 ± 6.22	−1.605	0.112	−0.352
Total score combined (0–54)^a^, mean ± SD	25.44 ± 9.93	32.12 ± 8.88	−3.233	**0.002**	−0.710

Note: p-values of < 0.05 are marked in bold

Results were analysed with independent samples t-test (Cohen’s d) and presented as mean, SD, and t- value

^a^Total possible range for SNAP-IV, scored best–worst

ADHD, attention deficit hyperactivity disorder; SNAP-IV, The Swanson, Nolan, and Pelham Rating Scale; SD, standard deviation

## Discussion

The principal findings of this study showed that, among children aged 6–14 years with ADHD, those without clinically relevant sleep problems after the sleep intervention reported significantly fewer insomnia symptoms and less tension compared to those who continued to have clinically relevant sleep problems. In addition, their parents rated the severity of attention deficit symptoms as lower. These results suggest that improvements in sleep are associated not only with better subjective sleep experiences but also with a reduced burden of ADHD symptoms as perceived by parents. However, no significant differences between the groups were found regarding self-reported life functioning or overall health-related quality of life, suggesting that broader aspects of daily functioning may be less directly influenced by sleep improvements in the short term.

The children with ADHD and without clinically relevant sleep problems tended to experience insomnia (as measured by ISI) to a lesser extent than children with ADHD and clinically relevant sleep problems. To the best of our knowledge, no studies have compared insomnia in children with ADHD based on the clinical cut-off by Parreira et al. [25], but Akinci et al. [2] found that children with ADHD, in comparison with controls without ADHD, reported more frequent problems with insomnia. Sciberras et al. [8] found five various sleep profiles in children with ADHD, with the insomnia profile being the most common, indicating that insomnia is a significant sleep problem in this group of children. More research is needed regarding insomnia and other sleep problems in children with ADHD and how the characteristics of sleep problems are conveyed in parent-reported instruments as well as in child-reported instruments.

The children without clinically relevant sleep problems in our study exhibited lower levels of the anxiety symptom ‘tension’ (as measured by short-STAI), while no significant differences were observed in other anxiety symptoms between the groups. In line with our results, Sciberras et al. [8] found that children with ADHD without sleep problems displayed anxiety symptoms to a lesser extent than children with ADHD in terms of the sleep profiles of insomnia, generalised sleep difficulties, and high bedtime resistance. Mayes et al. [34] demonstrated that children with ADHD, particularly those comorbid with anxiety or depression, often struggled with various sleep problems. The relationship between sleep problems and anxiety symptoms in children with ADHD warrants more research, particularly regarding how remission in sleep problems could potentially decrease anxiety levels.

There were no differences in life functioning (as measured by CORS) or health-related quality of life (as measured by EQ-5D-Y-3 L) between children without and with clinically relevant sleep problems in our study. This contradicts previous results presented by Sciberras et al. [8] where children with ADHD without sleep problems were reported to have fewer emotional problems than children with ADHD and sleep problems. Those results were, however, based on parental reports and not self-reported by the children, as in our study. Moreover, their assessment of emotional problems included similar aspects to those covered in our life functioning and health-related quality of life measures, but the questionnaires used were not the same. Yürümez & Kılıç [35]. assessed quality of life in children with ADHD (with and without sleep problems) and a control group of children without ADHD and found worse scores for quality of life for children with ADHD reported by both parents and children respectively. No analyses for quality of life were, however, performed between children with ADHD and with and without sleep problems. More studies are needed to elucidate the relationship between various levels of sleep problems, life functioning, and health-related quality of life in children with ADHD, as well as the accordance between self-reported and parental reported measures for these factors.

Parents of children without clinically relevant sleep problems in our study reported fewer ADHD symptoms (as measured by SNAP-IV) on the attention deficit scale compared to parents of children with clinically relevant sleep problems. This aligns with the findings of Sciberras et al. [8], who demonstrated that children with ADHD without sleep problems had fewer problems with ADHD symptoms compared to children with ADHD and sleep problems. Yin et al. [36] further highlights the relationship between increasing ADHD symptoms and increasing sleep problems. The findings from our study and from previous literature underscores the importance of considering sleep in the evaluation and management of ADHD symptoms in children, as sleep problems may exacerbate ADHD-related behaviour and impairments.

Taken together, these findings highlight the central role of sleep in the health of children with ADHD. While children without significant sleep problems reported better sleep and fewer ADHD symptoms, those who continued to struggle with sleep represent a vulnerable group in need of targeted interventions. Addressing sleep difficulties in children with ADHD may be an important pathway not only for improving sleep itself, but also for reducing ADHD symptom severity and supporting overall well-being.

### Strengths and limitations

The study’s cross-sectional design allowed for a comprehensive overview of the participants’ health-related factors, aiding comparisons between different groups of children with ADHD, without or with clinically relevant sleep problems. The use of established cut-off criteria for clinically relevant sleep problems enhanced classification precision within the selected population. Thorough participant recruitment based on specific criteria ensured a homogeneous group, strengthening validity. Established measurement instruments facilitated multidimensional assessment, while robust data analysis ensured reliability. However, the limitations included the inability to capture temporal changes or establish causality with the current study design. There is a risk of Type II error and that differences that exist may have been missed due to the relatively small sample. Moreover, multiple independent comparisons were performed in our study, which increases the risk of false positive results, especially for p-values above 0.01. Reliance on subjective measurement instruments may have increased the risk of bias and the age differences might have affected the result. Where the data collection procedure is concerned, there seems to be a discrepancy between child-rated and parent-rated outcomes in terms of severity of sleep problems. For example, CSHQ is not validated for use in children older than 12 years [23, 24], which impacts on the concordance between parent and child-reported sleep. Sociodemographic and parental factors might affect the outcome and generalizability, however, the study has a relatively balanced sample which could improve the understanding of the relationship between sleep problems and health variables in this population. Another limitation is that restless legs syndrome and sleep disordered breathing that in previous studies have shown associations with ADHD were not assessed in the current study.

## Conclusions

Our study highlights a potential link between a lower level of self-reported insomnia and of the anxiety symptom ‘tension,’ and fewer parent-reported ADHD symptoms for attention deficit in children with ADHD without clinically relevant sleep problems compared to their counterparts with clinically relevant sleep problems. No differences in self-reported life functioning and health-related quality of life were found between children without and with clinically relevant sleep problems. Our findings indicate that better sleep in children with ADHD improves their well-being and ADHD-related symptoms which may affect their overall health. Since better sleep has the potential to reduce ADHD symptoms, an increased focus on sleep when meeting children with ADHD may be of value. These results may be used in clinical practice to promote a holistic approach regarding management of sleep problems in children with ADHD. These findings require validation in larger groups of children with ADHD and various levels of sleep problems. Furthermore, longitudinal studies are necessary to fully comprehend the long-term impact of sleep problems and various health-related factors in this cohort. Investigations of long-term effects following targeted interventions are also of importance.

## Data Availability

The datasets generated during and/or analyzed during the current study are not publicly available due to legal/ethical reasons but are available from the corresponding author on reasonable request.

## Abbreviations

ADHD: Attention-Deficit/Hyperactivity Disorder
CAMHS: Child and Adolescent Mental Health Service
CORS: The Child Outcome Rating Scale
CSHQ: Children’s Sleep Habits Questionnaire
CSHQ-SWE: Swedish version of the Children’s Sleep Habits Questionnaire
IQR: Interquartile range
ISI: The Insomnia Severity Index
Q: Quartile
SD: Standard deviation
Short-STAI: The short form of State-Trait Anxiety Inventory
SNAP-IV: The Swanson, Nolan, and Pelham Rating Scale
